# Evidencing the impact of cancer trials: insights from the 2014 UK Research Excellence Framework

**DOI:** 10.1186/s13063-020-04425-9

**Published:** 2020-06-05

**Authors:** Catherine R. Hanna, Lauren P. Gatting, Kathleen Anne Boyd, Kathryn A. Robb, Rob J. Jones

**Affiliations:** 1 CRUK Clinical Trials Unit, Institute of Cancer Sciences, University of Glasgow, 1053 Great Western Road, Glasgow, G12 OYN UK; 2 Institute of Health and Wellbeing, University Of Glasgow Gartnavel Royal Hospital, Admin Building, 1st Floor, 1055 Great Western Road, Glasgow,, G12 0XH UK; 3 Institute of Health and Wellbeing, University Of Glasgow, Health Economics and Health Technology Assessment, 1 Lilybank Gardens, Glasgow, G12 8RZ UK

## Abstract

**Introduction:**

An impactful clinical trial will have real-life benefits for patients and society beyond the academic environment. This study analyses case studies of cancer trials to understand how impact is evidenced for cancer trials and how impact evaluation can be more routinely adopted and improved.

**Methods:**

The United Kingdom (UK) Government allocates research funding to higher-education institutions based on an assessment of the institutions’ previous research efforts, in an exercise known as the Research Excellence Framework (REF). In addition to each institution’s journal publications and research environment, for the first time in 2014, allocation of funding was also dependent on an evaluation of the wider, societal impact of research conducted. In the REF2014, impact assessment was performed by evaluation of impact case studies. In this study, case studies (*n* = 6637) submitted by institutions for the REF2014 were accessed and those focussing on cancer trials were identified. Manual content analysis was then used to assess the characteristics of the cancer trials discussed in the case studies, the impact described and the methods used by institutions to demonstrate impact.

**Results:**

Forty-six case studies describing 106 individual cancer trials were identified. The majority were phase III randomised controlled trials and those recruiting patients with breast cancer. A list of indicators of cancer trial impact was generated using the previous literature and developed inductively using these case studies. The most common impact from a cancer trial identified in the case studies was on policy, in particular citation of trial findings in clinical guidelines. Impact on health outcomes and the economy were less frequent and health outcomes were often predicted rather than evidenced. There were few descriptions identified of trialists making efforts to maximise trial impact.

**Discussion:**

Cancer trial impact narratives for the next REF assessment exercise in 2021 can be improved by evidencing actual rather than predicted Impact, with a clearer identification of the beneficiaries of cancer trials and the processes through which trial results are used. Clarification of the individuals responsible for performing impact evaluations of cancer trials and the provision of resources to do so needs to be addressed if impact evaluation is to be sustainable.

## Introduction

The success of a modern cancer trial should not be determined solely by the trial results or the impact factor of the journal of publication. In addition, this success should be based on the real-life benefits that the trial makes to patients and society. Several institutions that fund or perform cancer trials, including Cancer Research UK, the Institute of Cancer Research and the Dana-Farber Cancer Institute, have formally endorsed the San Francisco Declaration on Research Assessment [1]. This declaration states that the evaluation of scholarly output should focus on meaningful benefits arising from research rather than narrow, quantitative metrics.

Cancer trials attract substantial investment from public and private funding. In 2019, the National Cancer Institute received over US$6 billion from Congress to fund cancer research, with over US$800 million spent on clinical trials [2, 3]. Cancer Research UK, which is the single largest funder of cancer research in the United Kingdom (UK), spent £546 million on cancer research in 2018/2019 [4], has pledged £45 million specifically to its eight clinical trials units and [5] recruits over 25,000 patients to its clinical trials per annum [6].

In order to show accountability for these investments and to demonstrate to the public that money is invested wisely, it is crucial to show that academic outputs from cancer trials are leading to broader changes and benefits to society. These benefits are commonly referred to as the impact of research. The UK Higher Education Funding Council for England states that impact is *‘*an effect on, change or benefit to the economy, society, culture, public policy or services, health, the environment or quality of life, beyond academia’ [7].

Demonstrating the real-life impact of cancer trials can illustrate to patients and the public the value of participating in clinical trials. Outlining to healthcare managers the benefits that cancer trials bring to the health system may increase the time allocated to clinicians for trial recruitment. Demonstrating to funders that trials are impactful and identifying which types of trial have most impact means that funders can prioritise clinical trial investment. This is important because there is an opportunity cost that accompanies the decision to develop and perform one trial rather than another, due to the limited pool of patient volunteers and administrative support available. For example, Carlisle et al. [8] have demonstrated that clinical trials of cancer monotherapy conducted in the post-regulatory approval setting contribute less to subsequent drug approval and clinical guidelines than trials conducted for approval purposes. This is despite an at least equivalent burden for patients in terms of numbers needed for recruitment and the proportion who experience serious adverse events related to trial treatment. Only by understanding the impact of previous trials can funders, policy-makers and clinicians design, prioritise and invest in increasingly impactful trials in future.

Although the evaluation of research quality is not new, the assessment of research impact is a more recent phenomenon. The UK Government allocates research funding to higher-education institutions based on an assessment of the institutions’ previous research efforts. This allocation has traditionally focussed on an assessment of institutions’ journal publications and the research environment and prior to 2014 (1986–2008), was known as the Research Assessment Exercise. For the first time in 2014, allocation of funding was also dependent on an evaluation of the wider, societal impact from research. The name of the assessment was changed to the Research Excellence Framework (REF), and, in the exercise conducted in 2014 (REF2014), assessment of research impact was performed by evaluation of case studies. Impact case studies are narratives written by the institutions to describe the downstream effects that the institution perceive to represent the wider, societal impact related to their research, that is external to academia. The REF was piloted in the UK in 2010, formally employed in 2014, and the next assessment is due in 2021. Through this exercise the government allocates over £2 billion per annum to higher-education institutions and in 2021, impact case studies will attract an even greater proportion of funds (25%) compared to 2014 (20%). Partly because of the REF, the ability of UK universities to demonstrate that their research has led to real-life, tangible benefits to society, has become a major determinant of core income and status for these institutions. Other countries, such as Australia and Canada, are now (re-) investigating the use of impact assessment as part of their national evaluation frameworks [9, 10].

Several authors have reflected on how universities evidenced the impact of their research in the REF2014. Greenhalgh and Fahy [11] outlined 14 types of impact evidenced by higher-education institutions in 162 impact case studies submitted to the REF2014 community-based disciplines’ panel. They found that an influence on guidelines was most commonly described, followed by impact on informing policy change and changes in clinical or public health practice. Chowdhury, Koya and Philipson [12] reviewed 363 case studies in six disciplines from either top-ranking or bottom-performing institutions in the REF2014 and identified variables that predicted the average REF scores received by the institutions. For 92 case studies submitted under the discipline of Clinical Medicine, the number of publications in highly cited journals was the variable most consistently associated with higher REF scores. These authors also used automated word frequency analysis to identify themes of research submitted under different disciplines. For clinical medicine, these included oncology, paediatrics, genetics, diabetes and heart disease research. Terämä et al. [13] used computational text-mining of the REF2014 case studies to understand how higher-education institutions interpreted impact. By analysing 6637 case studies, six classes of impact were identified (1 – Education, 2 – Public engagement, 3 – Environment and energy solutions, 4 – Enterprise, 5 – Policy; 6 – Clinical uses) and the class of impact described differed according to discipline. Similarly, a review of the REF2014, commissioned by the Higher Education Funding Council for England, discovered that frameworks and taxonomies of impact were often context specific [14].

The aim of this paper was to use the REF2014 case studies to understand how higher-education institutions evidenced the impact of their cancer trials. Such an understanding will allow reflection on if, and how, impact assessment for cancer trials can be performed outside the context of the REF, and how impact evaluation can improve, both for REF2021 and beyond.

The objectives were:

- To identify cancer trials included by higher-education institutions in the REF2014 case studies
- To quantify and explore the characteristics of these trials and the types of impacts they were claimed to have had
- To identify the types of evidence used by higher-education institutions to substantiate those claims of impact
- To identify any examples of researchers or research users making active attempts to maximise impact

## Methods

### Data collection

The REF2014 impact case studies are stored online and are publicly available via the Research Excellence Framework 2014 website [15]. A search of the non-confidential case studies was performed by combining the terms ‘cancer’ and ‘trial’ in the website search function [15]. This search function identified case studies that included these words in any part of the submission (title, main text or references). The case studies identified were read in full and the application of inclusion and exclusion criteria at this stage allowed the selection for final analysis. Inclusion criteria required that the case study focussed on the impact of adult (aged 16 years or over) clinical trials that prospectively recruited patients with a diagnosis of malignancy, or individuals without a known diagnosis but where the aim of the trial was to investigate the development of, diagnosis or screening of cancer. All stages of cancer and clinical trials of all phases were included. Impact case studies were excluded if they described paediatric cancer trials (age < 16 years) and/or if clinical trials were mentioned but were not the focus of the case study.

### Data analysis

**Fig. 1:**
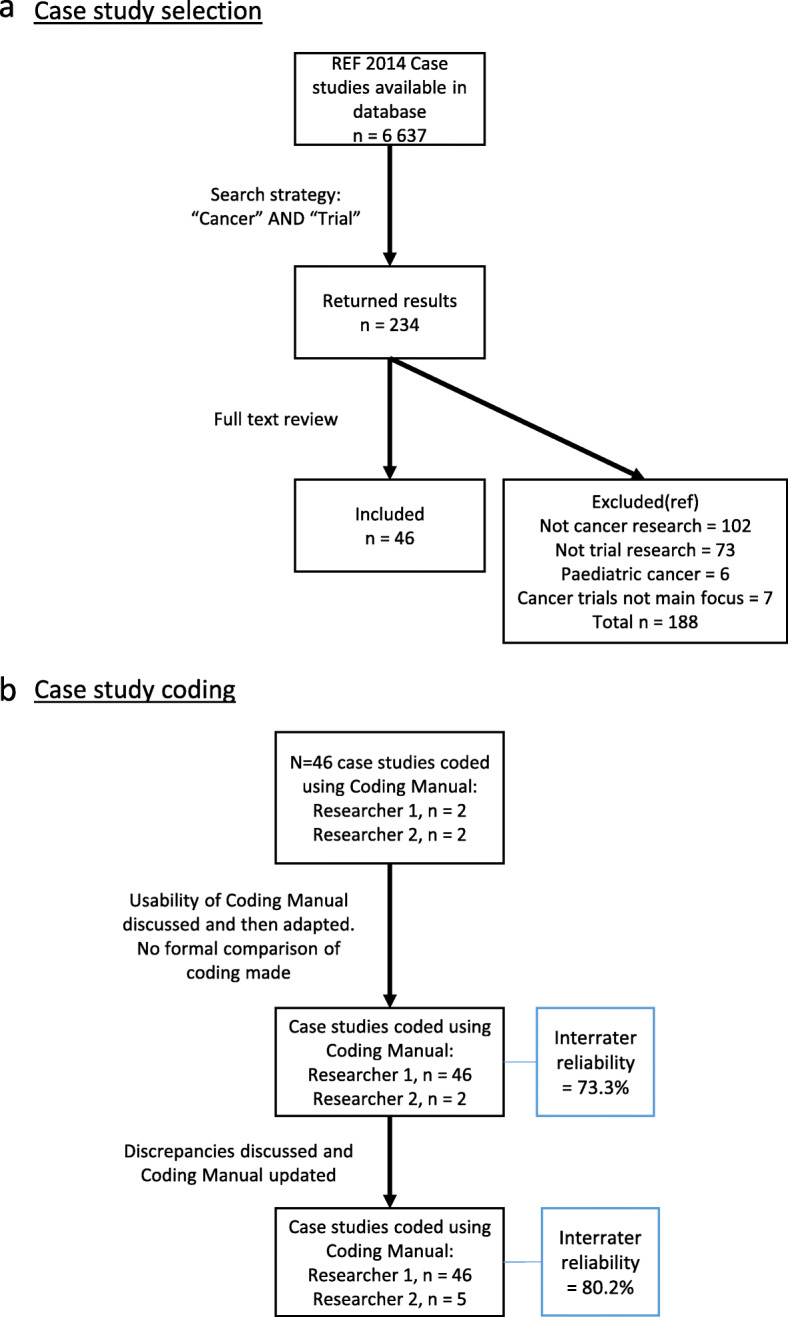
**a** Case study selection. **b** Case study coding

Manual content analysis of the case studies meeting these criteria was performed [16]. The initial coding manual was based on previous literature [11, 17–19], collected descriptive information about the case studies and cancer trials, and contained pre-defined categories of impact that were identified from a systematic review (unpublished). Supplementary material 2 explains in more detail how these categories of impact were identified. The manual was developed iteratively through three stages by two researchers (CH and LG) to better reflect the specific context of cancer trial impacts. For a detailed outline of the coding process, see Fig. 1b. This iteration included the inclusion of specific examples, often referred to as indicators [12], of how higher-education institutions evidenced impact within each categories. The second reviewer (LG) coded a randomly selected sub-sample of the case studies to assess coding validity. The final inter-coder reliability estimate for this was 80.2%.

In Part 1 of the coding manual (Supplementary material 1) the following information was recorded: (1) the institution responsible for the submission; (2) the Unit of Assessment and (3) the Summary Impact Type. The Units of Assessment are 36 subject areas, each with its own REF expert review panel. The Summary Impact Types are eight categories of impact, assigned to each case study by text analysis after submission to the REF. These categories are technological, economic, health, political, legal, cultural, societal and environmental [13]. For the clinical trials identified, the following key characteristics were extracted: (1) name; (2) phase of the trial; (3) type of cancer investigated; (4) focus of the trial (screening, diagnosis and treatment, other); (5) journal of publication cited in the case study; (6) category of funder; (7) primary endpoint and (8) whether the primary endpoint was met. For the purposes of the final characteristic, trials were marked as positive if they met their pre-specified primary endpoint with statistical significance. For non-inferiority trials, if the experimental arm of the trial was deemed to be statistically non-inferior than the control arm at the level of significance pre-defined by the trialists, this was considered a positive result. For earlier-phase trials such as phase I trials focussing on safety, if, for example, the authors set out to find a recommended phase II dose of a novel drug, and this was achieved and reporting in the trial findings, this was considered as having a positive result.

Part 2 of the coding manual (Supplementary material 1) captured the following information for each impact case study: (1) all categories of impact described; (2) examples of dissemination and knowledge transfer of trial information and results; (3) methods used by institutions to evidence impact; (4) clinical guidelines cited and (5) examples of when researchers or research users acted to enhance trial impact [20]. Dissemination and knowledge transfer describe the communication of trial information or results to stakeholders. This information was collected by reading and manually coding the ‘Details of Impact’ section of each case study using Nvivo version 12.1 (2018). The pre-defined categories of cancer trial impact were (i) ‘New knowledge and immediate research outputs’, (ii)‘Capacity building for future research ’, (iii) ‘Policy and guidelines’, (iv)‘Health sector services and clinical practice’, (v) ‘Improved health for patients and public’, (vi)‘Economic’ and (vii) ‘Social and cultural’ impact. A distinction was made between those case studies in which institutions’ described potential health impacts versus those in which the institution evidenced health improvements that had actually occurred; for example, through the use of audit data or epidemiological studies.

## Results

### Impact case studies

Out of 6637 publicly available REF2014 impact case studies, 234 were returned as potentially relevant based on the combined word search of “Cancer” AND “Trial”. On reading the full submissions of these 234 case studies, 46 met the pre-defined inclusion criteria. Figure 1a presents the search results in a PRISMA style diagram [21] and details the reasons for exclusion. The REF Unit of Assessment, Summary Impact Type and name of institutions responsible for the submission for each case study are shown in Table 1.

**Table 1:** Key characteristics of included case studies and trials

Case studies (*n* = 46)	Number	Percentage^c^
*Research Excellence Framework (REF) impact type*^a^		
Health	37	80%
Technological	8	17%
Political	1	2%
*REF Unit of Assessment*^b^		
Clinical Medicine	38	83%
Public Health, Health Services and Primary Care	4	9%
Allied Health Professions, Dentistry, Nursing and Pharmacy	2	4%
Psychology, Psychiatry and Neuroscience	1	2%
Biological Sciences	1	2%
Trials (*n* = 110)^d^		
*Trial focus*		
Treatment of cancer or its side effects	97	88%
Screening	6	5%
Diagnosis	4	4%
Other (e.g. large observational trial to investigate cancer incidence)	3	3%
*Phase of trial*		
I	17	15%
II	14	13%
III	75	68%
Unknown/other	4	4%
Higher-education institution (*n* = 19)		
University College London	9	20%
Institute of Cancer Research	4	9%
University of Leeds	4	9%
University of Manchester	4	9%
Queen Mary University of London	4	9%
Imperial College London	3	7%
University of Cardiff	2	4%
University of Edinburgh	2	4%
University of Glasgow	2	4%
University of Nottingham	2	4%
University of Oxford	2	4%
University of Birmingham	1	2%
University of Bradford	1	2%
University of Bristol	1	2%
Cardiff University	1	2%
University of Cambridge	1	2%
King’s College London	1	2%
Newcastle University	1	2%
University of Southampton	1	2%
Diagnoses of patients recruited to the included clinical trials (*n* = 110)^d^		
Breast	38	35%
Gastrointestinal (lower)	15	14%
Haematological	15	14%
Urological	13	12%
Gynaecological	10	9%
Thorax	8	7%
Central nervous system	4	4%
Head and neck (including thyroid)	3	3%
Multiple cancer types	3	3%
Gastrointestinal (upper)	1	1%
Main source of clinical trial funding (*n* = 110)^d^		
Industry only	33	30%
Charity and Research Council/Government/University	19	17%
Research Council/Government/University only	16	15%
Unknown	14	13%
Charity and Industry	13	12%
Charity, Industry and Research Council/Government/University	8	7%
Charity only	7	6%

^a^The Summary Impact Types are eight categories of impact, assigned to each case study by text analysis after submission to the REF. These categories are technological, economic, health, political, legal, cultural, societal and environmental

^b^The Units of Assessment are 36 subject areas, each with its own REF expert review panel. These subject areas are divided into four main panels which group similar research disciplines. For example, Panel A includes Clinical Medicine and Biological Sciences, Panel B: Chemistry and Physics, Panel C: Law and Economics, and Panel D: History, Classics and Languages. Each discipline listed within these main panels represents one Unit of Assessment

^c^May not add to 100% due to rounding

^d^Each clinical trial (*n* = 106) was counted for each individual case study in which it was mentioned (a total of 106 trials mentioned in separate case studies 110 times)

### Characteristics of the cancer trials identified

The number of trials specifically cited in each case study ranged from 1 to 7. Overall, 106 individual trials were referenced 110 times. The majority of trials identified (68%) were phase III randomised clinical trials and most trials focussed on the treatment of cancer (88%); trials investigating screening and diagnosis were much less common at 5% and 4%, respectively. A large proportion recruited patients with a diagnosis of breast cancer (35%) (Table 1). The Arimidex, Tamoxifen, Alone or in Combination (ATAC) trial [22] was discussed in five separate case studies by four universities [23–27]. The ATAC trial investigated the efficacy of an orally administered aromatase inhibitor compared to an orally administered anti-oestrogen for the adjuvant endocrine treatment for postmenopausal women with hormone-receptor-positive, localised breast cancer. When used by the same university, one case study focussed on the impact on clinical practice change worldwide and the sales for the drug company responsible for the production of the aromatase inhibitor [25]. The second focussed on subsequent research by the same university in response to knowledge generated from the ATAC trial around drug-associated bone loss [26]. Impacts described in the other three ATAC trial case studies included the provision of tumour specimens for translational research and investigation of novel biomarkers [27], citation of the trial results in guidelines with subsequent impact on clinical practice and breast cancer relapse [24, 27]. The Prostate Testing for Cancer and Treatment (ProtecT) trial [28], which was still recruiting at the time of REF2014 submissions, was described by two universities as an example of their work [29, 30]. Both institutions outlined the collaborative approach to designing and performing this trial and the impact that the background work for the trial contributed to the concept of active monitoring for men with prostate cancer and on providing evidence to support a government decision not to introduce prostate cancer screening.

**Fig. 2:**
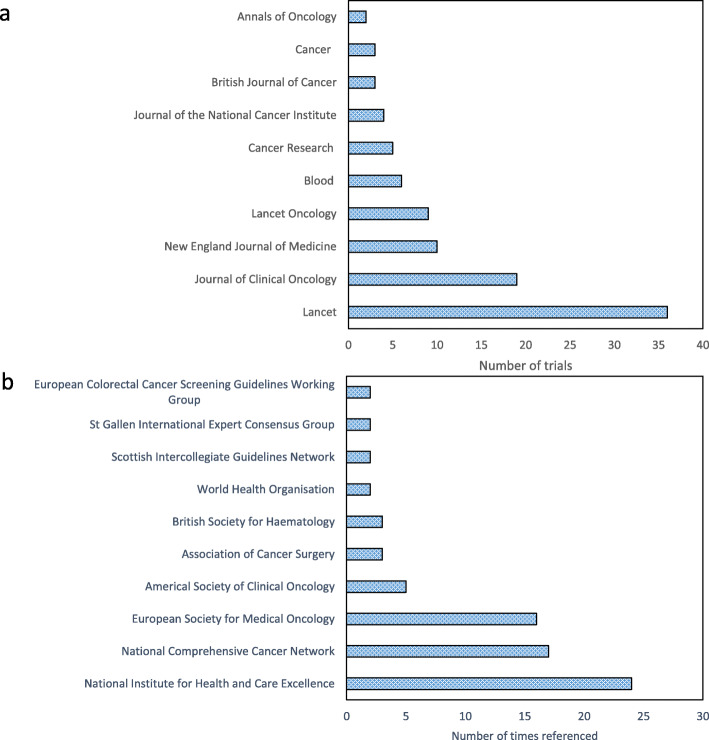
**a** Ten most common journals of trial publication. **b** Ten most frequently referenced national or international clinical guidelines

As shown in Table 1, there were often collaborative funding streams for these clinical trials from industry, the charity sector and government-led research councils. Figure 2a shows that the journals of publication included both cancer-specific journals and those aimed at a more generic clinical readership. The most common primary outcomes evaluated were overall or cancer-specific survival (18%; 20/110) or a measure of disease recurrence or progression (18%; 20/110). Several trials used a co-primary endpoint (16%; 18/110). Although most trials (78%; 86/110) met their primary endpoint, one fifth of trials (20%; 22/110) did not and, for a minority of the trials (2%; 2/110) this was unclear.

### Categories of cancer trial impact

The frequency with which different categories of impact were identified in the case studies are shown in Table 2. Most case studies (93%) described the impact of cancer trials on policy, and in particular, the citation of trial results in national or international clinical guidelines. A list of the ten clinical guidelines in which these trials are most cited is in Fig. 2b. None of the case studies referred to social or cultural impacts of clinical trials. One case study did explain that a clinical trial had changed ‘culture and behaviour’, but on reading the narrative this was coded as a change in the prescribing practice of clinicians [31]. Another case study [32] discussed differences in cancer screening uptake between different socioeconomic groups which was partly identified by a clinical trial and has led to funding for a future trial to investigate and tackle this problem. There is potential for this subsequent trial to have substantial social impact if it successfully identifies ways to address this screening uptake imbalance.

**Table 2:** Categories and sub-categories of impact and frequency identified within all 46 case studies

Category/sub-category^a^	Case study references for this sub-category (number)	Case study references for this sub-category (percentage)
1. New knowledge and immediate research outputs	39	85%
1.1 New knowledge generated directly from clinical trial	38	83%
1.2 New knowledge from clinical trial has contributed to a secondary analysis, e.g. systematic review or meta-analysis	3	7%
2. Capacity-building for future research	24	52%
2.1 Clinical trial has contributed to the development (or intentional ceasing of the development) of further research, clinical trials and researchers	19	41%
2.2 Clinical trial has led to collaboration and/or data sharing	3	7%
2.3 Clinical trial has led to training of future clinicians and researchers	5	11%
2.4 Clinical trial has led to innovation and novel infrastructure (other than health-service related), e.g. the development of a novel technique or tool by a commercial company	4	9%
3. Policy and guidelines	43	93%
3.1 Clinical trial has influence policy agenda setting	7	15%
3.2 Clinical trial has led to a treatment approvals (e.g. drug, device, procedure licensing or marketing approval)	15	33%
3.3 Clinical trial contributed to clinical guidelines	39	85%
3.4 Clinical trial contributed to other public policy, e.g. government policy	6	13%
3.5 Clinical trial has provided justification of the implementation of existing policy	4	9%
4. Health sector (Health service)	16	35%
4.1 Clinical trial has influenced/benefitted health-service delivery	16	35%
Health sector (Clinical practice)	37	80%
4.2 Clinical trial has changed clinical practice and actual clinical practice has been evaluated	19	41%
4.3 Clinical trial has changed clinical practice and potential or estimated clinical practice has been evaluated	30	65%
5. Improved health for patients and public	32	70%
5.1 Clinical trial has contributed to improved health for patients (other than those in the trial) and actual health changes have been evaluated	7	15%
5.2 Clinical trial has contributed to improved health for patients (other than those in the trial) and health changes have been estimated	29	63%
6. Economic impact	25	54%
6.1 Clinical trial has led to direct cost savings for the health service	12	26%
6.2 Clinical trial has shown benefit of a diagnostic or management strategy that is cost-effective	8	17%
6.3 Clinical trial has led to measured or estimated benefits for the macro economy, e.g. sales of drug for a pharmaceutical company, setting up a new spin-off company	10	22%
6.4 Clinical trial has led to measured or estimated benefits to the macro economy from a healthy workforce, e.g. patient returning to work earlier	1	2%

^a^The sub-categories are not mutually exclusive and several sub-categories may be coded for each case study. The percentage indicators give the percentage of the case studies in which this category or sub-category was identified

### Dissemination and knowledge transfer

Overall, half (50%, 23/46) of case studies mentioned at least one type of dissemination or knowledge transfer. These examples were divided into a description of the publication of trial results in an academic journal (20% of case studies; 9/46), citation of the results publication in other academic articles (7%; 3/46) or other methods of communication (35%; 16/46) such as reports in the lay or social media, patient-facing websites and conference presentations.

### Methods of evidencing cancer trial impact

Common methods used by higher-education institutions to evidence the cancer trial impacts that were identified included: (1) identification of citations of trial publications in policy documents (78%; 36/46); (2) interrogation of real-life patient- or population-level data on clinical practice or health-service use (52%; 24/46); (3) the use of expert or user testimony (30%; 13/46) and (4) surveys (both quantitative and qualitative) (15%; 7/46). Interestingly, testimonies were only from researchers and funders, with none from policy-makers or patients. Although many (70%; 32/46) case studies described the impact that cancer trials had on changing health outcomes (Section 5 of the coding manual, Supplementary material), only seven (15%) described an actual, rather than predicted or estimated, change in health of patients (Section 5.1 of the coding manual) (Table 2). Several (39%; 18/46) case studies specifically quoted the monetary value of the funding linked to the research described in their case studies, totalling approximately £90 million. None incorporated this monetary value in an estimation of the economic return on research investment.

### Researchers and research users enhancing cancer trial impact

A minority (15%; 7/46) of case studies mentioned that researchers actively enhanced the impact of a clinical trial. Examples included researchers interacting with policy-makers to give advice on how to pilot implementation of clinical trial findings [33] and researchers making efforts to ensure that trial findings are presented in the lay media, health blogs and charity websites [31]. There was also an example of researchers training clinicians in the selection of patients who would benefit from radiotherapy treatment that had been developed in the context of a clinical trial [34]. The submitting institution explained that these actions help to ensure implementation of trial findings and improved uptake of this radiotherapy treatment in the UK. There was one example of when a research user enhanced the impact of a cancer trial. This occurred when a patient used the results from a cancer trial to lobby the UK government to fund a novel drug to treat breast cancer for treatment of patients within the UK [35]. Overall, the fact that there is a limited number of these examples does not imply that researchers or research users did not play an important role in the promotion, implementation and wider impact of cancer trial findings, but if this did occur, it was not identified by universities as an important part of their impact narrative within these case studies.

## Discussion

There have been prior reviews of the REF2014 case studies [19, 36–39], but this is the first analysis that focuses specifically on cancer research or clinical trials. This study shows that UK universities recognise cancer trials as impactful research undertaken at their institutions. Nineteen (12%) out of 154 institutions participating in the REF2014 submitted 46 case studies that specifically focussed on cancer trials. Most of the higher-education institutions were Russell Group Universities (89%; 16/19) [40], a self-selected association of 24 leading public research universities in the UK, whose member institutions submitted 68% of the highest-ranked (4* outstanding) case studies in the REF2014 [41]. The relatively small number of universities submitting cancer trial case studies implies that this type of research is concentrated at specific locations. Over half (54%) of the case studies described the impact of more than one trial, raising the question of whether it is feasible to expect a single trial, rather than a combination of trials or a programme of trials’ research, to lead to tangible impacts on patients and society. Lastly, several universities described the impact of the same trial, illustrating the collaborative approach adopted at those institutions.

Trials recruiting patients with breast cancer constituted over a third of the included trials; a much greater proportion than those recruiting patients with, for example, lung cancer (7%). Although breast cancer is the most common cancer (15% incidence) in men and women combined in the UK [42], lung cancer has the highest mortality rate and accounts for over one fifth of all cancer deaths (2017) [43]. Skin cancer, including melanoma, germ-cell cancer and sarcoma were in the coding manual but no trials were identified that solely included patients with these diagnoses. It is likely that, rather than accurately reflect the relative burden of these cancers in the UK [44], these case studies reflect the landmark trials that reported results within the assessment REF2014 eligible period (1993–2014). There were no trials reporting the benefits of immunotherapy, widely regarded as a major recent advance in cancer treatment. Again, it is likely that this reflects the publication dates of key trials investigating the novel immunotherapies and it will be interesting to analyse whether these trials are in the case studies submitted to the REF2021. The ten journals in which the clinical trials described in these case studies were most frequently published all have a Journal Impact Factor over 5 and the top three have an Journal Impact Factor above 25 [45]. This supports the findings from Chowdhury, Koya and Philipson [38] that, although not an article-level metric and not a measure of impact, the research outputs underlying REF2014 impact case studies were often published in journals with a high average citation count.

Higher-education institutions did not exclusively use clinical trials that met their pre-specified primary endpoints in these case studies as examples of impactful research. For example, the LIBERATE trial [46] closed early because an increase in breast cancer recurrence was found to occur in patients being managed with hormone replacement therapy to treat symptoms following cancer treatment. The submitting university argued that the impact of this trial was a change in guidelines to prevent subsequent use of hormone replacement therapy for this group of patients. Another example was the FOCUS2 trial [47], which tested the optimal treatment for elderly and frail patients with metastatic colorectal cancer. Although the trial did not meet its primary endpoint, it demonstrated the feasibility of recruiting patients from an often under-researched patient cohort. It also provided important information around toxicity and quality of life that has subsequently been cited in clinical guidelines and changed clinical practice. This demonstrates that the pathway to impact is not solely dictated by practice-changing trials, but that practice-affirming trials may be impactful by preventing harmful variation in practice [48, 49].

The fact that some institutions used early phase trials as standalone examples of impactful research shows that robust examples of real-life impact do not only emerge from large, later-phase trials. As an example, a portfolio of trials which demonstrated the safety, optimal dosing and blood-brain-barrier penetration of a drug for patients with brain tumours, led to both direct (licensing of the drug) and indirect impacts (a phase III trial performed at another institution, subsequent introduction of the drug into routine practice and increased revenue for the pharmaceutical company) [50]. Another case study described the impact of early phase trials investigating the use a targeted treatment for patients with *BRCA*-associated breast and ovarian cancer. The significant improvement in outcomes for this sub-group of patients meant that these trials directly influenced international guidelines for genetic testing and led to further research investment and collaboration with industry for that institution. Submission guidelines indicate that examples of indirect impact will be welcomed in the REF2021 [51, 52].

The REF2021 expects that institutions will describe the process through which impact occurs, including, where possible, evidence of dissemination leading to impact. Ensuring transparency by informing patients and the public of the results of research, in particular clinical trials, is one of the UK’s Health Research Authority’s major priorities for ethical research practice [53]. It was, therefore, encouraging that some institutions in REF2014 described methods of knowledge transfer other than journal publication. In contrast, although there were examples of researchers or research users enhancing trial impact, these were identified in only a selection of case studies. Improved and more frequent descriptions of how trialists engage with end users of clinical trials to maximise timely trial impact could help submitting institutions to better demonstrate the process through which impact occurs in the REF2021. Finally, there was a small number of case studies evidencing actual impact that has occurred using methods such as the analysis of national audit data [34] or quantification of drug sales to indicate practice change [27, 54], or referencing epidemiological studies to show improved health outcomes [31]. Describing actual impact presents significant challenges in terms of timelines and planning, but gives a much stronger indication of the real-life benefits from cancer trials compared to estimations of potential impacts and it is likely that the former will be viewed favourably in the REF2021 [51].

Reflecting on the findings of this study provides optimism towards the more routine adoption of cancer trial impact evaluation, but also highlights challenges going forward. It is reassuring to see that cancer trials, a type of applied scientific research, are having real-life benefits for patients. Looking at the narratives submitted by higher-education institutions it is clear that impact evaluation is a useful way to scrutinize and reflect on the merits of the vast amount of work and investment required to perform these trials and that institutions have been able to evidence this real-life impact. In addition, by paying careful attention to trial impact, it is likely that this will contribute to better research prioritisation in the future. What is less clear from this study is who should be responsible for performing these evaluations, and if there is an expectation on primary researchers, such as clinicians, statisticians and health economists, to adopt this role, or if a new breed of researchers will emerge to answer this call. Impact assessment requires the utilisation of methods such as surveys, interviews and the analysis of large datasets, which are skills that may not be routinely utilised by cancer trialists. In addition, preparation of submissions to the REF2014 costs UK higher-education institutions £246 million, with £55 million spent on impact evaluation. This was a 133% increase from the Research Assessment Exercise in 2008 [55]. If the assessment of real-life cancer trial impact is to become a priority for the government and funders, provision of resources to perform such evaluations will need to be addressed either through core funding or specifically within clinical trial research grants.

Table 3 offers some suggestions of how to better evaluate, communicate and maximise cancer trial impact in the future. Whether trialists will hold responsibility for impact assessment or not, articulating the expected impact of a trial during the design phase, in collaboration with patients, will make subsequent impact evaluation easier and may focus trial design to address unmet needs. Tracking the impact of clinical practice on a national level will require access to routinely collected healthcare data, with sufficient granularity to make meaningful claims regarding the evidencing of impact and the identification of barriers to impact. Although the REF2014 website offers a list of impact case studies from many disciplines, it would be more useful if future impact narratives relating to cancer trials were to be publicised on more clinician- and patient-friendly platforms. Opening dialogue about research impact in a way that resonates with funders and trialists may encourage trial design with a focus on longer-term outcomes, such as changes in health or clinical practice, in a way that actually makes trials more impactful. The coding manual used in this study (Supplementary material 1) may offer a starting point for trialists to consider how they could embed impact evaluation into the routine review of their clinical trial outputs.

**Table 3:** Suggestions for evaluating, communicating and maximising cancer trial impact

	Recommendations:	Target group^a^
Evaluating the impact of cancer trials	• Educate trialists to anticipate the types of data required to evaluate impact and the collection methods to acquire this data. For example, surveys of current practice, accessing routine prescribing datasets	HEIs Funders
	• Use indicators of cancer trial impact (for example, those in supplementary material) to more routinely identify the wider impacts of future trials and to describe the impact of completed trials	Trialists CTUs HEIs
	• Assess how cancer trial results are used by decision-makers. This will create a narrative of the pathways through which impact occurs (direct and indirect). This process may uncover unexpected and less clearly defined impacts	Trialists CTUs Researchers HEIs
	• Identify examples of researchers or patients actively contributing to maximising trial impact	Trialists CTUs
	• Evaluate the impact of negative trials. Demonstrating impacts that do not rely on positive trial results will encourage funders and researchers to adopt a broader approach to clinical trial output assessment	Trialists CTUs
	• Provide funding and support for robust cancer trial impact evaluation	Funders HEIs
Communicating the impact of cancer trials	• Publicise cancer trial impact evaluations. Platforms for publicising evaluations could include patient-facing charity websites, CTU websites and clinical trial registries as well as more formal channels such as open access publications	Trialists CTUs HEIs Funders
Maximising the impact of cancer trials	• Incorporate impact assessment into the trial design process. This will generate ideas for researchers and CTUs of how they can take a more active role in maximising impact	Trialists CTUs
	• Continue to provide opportunities for trialists to engage with stakeholders, including patients, in the planning stages of clinical trial design to specifically explore the types of wider trial impacts that are important to stakeholders	Funders CTUs

*HEI* higher-education institution, *CTU* clinical trials unit

^a^The target group will also depend on who is performing the impact evaluation. As highlighted in the manuscript, there may be researchers, distinct from clinical trialists, who adopt the role of evaluating impact

There are several limitations to our analysis. Firstly, as with any review of the REF2014 impact case studies, these case studies were not specifically intended for this type of secondary analysis. Secondly, although having content validity for this study, the list of indicators of impact used to code these case studies (Supplementary material 1) will not be an exhaustive list of cancer trial impacts. In addition, for the purposes of this study we focussed on evaluating impact. Going forward, it would also be useful to make an assessment of the investment, both economic and non-monetary, into cancer trials. This would allow the impact of trials to be contextualised in terms of the investment provided from funders, and burden for patients from participating in these trials [8]. Finally, we used a binary assessment to indicate whether the primary endpoint was met for each trial. In future, this could be evaluated in greater detail by also looking at secondary endpoints or widening the evaluation to explore whether a trial met its objective to recruit sufficient patients to answer a clinical question.

Further research is required to understand which types of impact are important to patients and other stakeholders and the processes through which cancer trial impact occurs. It will be useful to repeat this exercise using the REF2021 case studies to identify which cancer trials conducted during 2000–2020 are regarded as most impactful by higher-education institutions, and to understand whether the methods of impact assessment have changed. Although not coded for the purposes of this study, a comparison of the dates of both the clinical trial and the impact evidenced would be useful. This would improve understanding of the time taken to achieve impact from UK cancer trials, which has previously been estimated to be in the order of 15 years for cancer research [56]. A better understanding of time lags specifically for cancer trials would provide insight into when an analysis of the return in cancer trial investment should ideally be performed and may identify opportunities to speed up impact in some scenarios [57].

This study should be helpful to institutions in any country who conduct cancer trials, and, in particular, in the UK as they currently prepare for their REF2021 submissions. It will also allow cancer trial funders to contextualise responses received when trialists describe the actual or potential impact of their work. The results should help conscientious cancer trialists and cancer trial units to consider how they can demonstrate the wider impact of their work to funders and patients. Ultimately, a better understanding and more routine adoption of impact assessment will provide the knowledge and vision required to ensure that we are conducting meaningful cancer trials research for patients.

## Supplementary information

**Additional file 1: Supplementary material 1:** Excel spreadsheet.

**Additional file 2: Supplementary material 2:** Diagram and Table.

## Data Availability

REF2014 impact case studies freely available online.
