# Supplementary material for: Evidencing the impact of cancer trials: insights from the 2014 UK Research Excellence Framework
Source: Trials. 2020 Jun 5;21:486. doi: 10.1186/s13063-020-04425-9 (PMC7275320; doi:10.1186/s13063-020-04425-9)
Supplement: Supplementary file 2 — Additional file 2: Supplementary material 2: Diagram and Table. [file 13063_2020_4425_MOESM2_ESM.docx]

Sustainability (I)

Communication/interactive processes (F)

Economic (I)

Social (H)

Social (B)

Multi-dimensional: health gains, economic, policy (F)

Collaboration (D)

Political (H)

Social (I)

Health and systems impact (G)

Health related and societal impact (G)

Scientific (H)

Environmental (I)

Broader economic impacts (G)

Influence on policymaking (G)

Policy impacts including clinical policies (F)

Primary research related impact (G)

Economic impacts (F)

Health service impact (D)

Industrial production (D)

Research activity, scientific production, and impact (D)

Public engagement, dissemination, culture and creativity (E)

Policy and public services impact (E)

Health and health sector benefits (E)

Economic (6)

Economic (C)

Innovative and economic impact (E)

Capacity building (B)

Capacity building (E)

Publication outlets (C)

New knowledge and immediate research outputs (including dissemination) (1)

Knowledge production and research targeting (E)

Social and cultural (7)

Social (C)

Cultural (C)

Technological (C)

Research networks (C)

Knowledge growth (C)

Economic (B)

Economic (A)

Health and health sector benefits (B)

Informing policies and product development (B)

Capacity building for future research (2)

Advancing knowledge (B)

Cultural (A)

Other

Social (A)

Scientific (A)

Health (4 and 5)

Policy impact (3)
